# Genomics for Weed Science

**DOI:** 10.2174/138920210790217972

**Published:** 2010-03

**Authors:** David Horvath

**Affiliations:** USDA-ARS, Bioscience Research Laboratory, 1605 Albrecht Blvd. Fargo, ND 58105, USA

## Abstract

Numerous genomic-based studies have provided insight to the physiological and evolutionary processes involved in developmental and environmental processes of model plants such as arabidopsis and rice. However, far fewer efforts have been attempted to use genomic resources to study physiological and evolutionary processes of weedy plants. Genomics-based tools such as extensive EST databases and microarrays have been developed for a limited number of weedy species, although application of information and resources developed for model plants and crops are possible and have been exploited. These tools have just begun to provide insights into the response of these weeds to herbivore and pathogen attack, survival of extreme environmental conditions, and interaction with crops. The potential of these tools to illuminate mechanisms controlling the traits that allow weeds to invade novel habitats, survive extreme environments, and that make weeds difficult to eradicate have potential for both improving crops and developing novel methods to control weeds.

## 1. GENOMICS TOOLS AND PURPOSE

Genomics is the study of the organization, evolution, and function of the genes and non-coding regions of the genome [1]. Various sub-disciplines of genomics focus on specific attributes of genome analysis. For example, functional genomics is the study of genes, function, and regulation and often centers on expression analysis tools such as microarrays (see below), and development of large scale reverse genetic resources such as libraries of thousands of individuals with characterized mutations in numerous genes [2], or identification of natural mutations from laboratory or wild populations [3]. Likewise, comparative genomics focuses on comparing the organization and sequences variation between species to obtain a better understanding of the evolution of genes and organisms [4]. Obviously there is overlap between sub-disciplines of genomics. For example the aim of “structural genomics” is to identify conserved protein domains [5]. Obviously such information would be relevant to functional and comparative genomics. Population genomics is similar to comparative genomics, but is focused on evolution of gene structure and function within and between closely related species [6]. The basic tools needed for genomic analysis include whole genome sequences, large scale mapping of genes, physical maps, bacterial artificial chromosome (BAC) libraries, collections of sequences of expressed genes (development of EST databases), methods to assess gene expression (at transcriptomic, proteomic and metabolomics level), as well as the databases for storage and computational programs needed to analyze and compare these huge datasets.

*De novo* whole genome sequencing is the holy grail of genomics for any organism, especially for non-model plants such as weeds [7]. The data generated from such a project not only assists in the identification of all real and potential genes within an organism, but also identification of *cis*-acting regulatory sequences (non-protein coding sequences surrounding genes that often contain the information needed for turning the genes on and off). Whole genome sequencing also provides a detailed physical map of gene order and distance. However, the cost of such a project makes it outside the reach of most researchers studying weeds and other non-model plants. Fortunately, the advent of new technologies such as long read 454 Titanium platforms, Solexa paired end pyrosequencing, and SOLiD 3 can produce large amounts of sequence data for significantly less cost than traditional Sanger sequencing methods. For example, the Titanium 454 FLX technology produces read lengths greater than 500 bases and can also generate ~ up to 600 Mb of sequence data for under $8,000. The various paired end read technologies such as SOLEXA can produce up to 20 gigabases (Gb) of random 75 – 100 base sequences (enough to provide a 10X coverage of the ~2Gb leafy spurge genome) for under $400 per Gb. Even greater cost reductions are achieved by the SOLiD 3 technology that boasts the generation of a Gb for under $300. These reduce the cost of such endeavors and bring the possibility of at least partial genome sequencing within the grasp of weed scientists [8]. However it should be noted that SOLEXA and SOLiD3 generate very short sequence reads, and it is untenable to use them for *de novo* genome sequencing. 

Although full genome sequencing is ideal, obtaining genomic sequences of gene-rich regions could provide a more economical approach to provide important regulatory sequences without wasting resources on sequencing vast stretches of repetitive DNA. There are several approaches that could be used to isolate gene-rich regions of the genome. These include preparing genomic libraries using bacterial strains that destroy hypermethylated DNA sequences [9]. Most actively transcribed genomic sequences are hypomethylated and thus would be protected in the bacterial strains. Another possibility would be to enrich the genomic DNA for low copy number sequences. This has been done by CoT hybridization followed by separation of the DNA fragments on hydroxyapetite columns [10]. A final possibility lies in novel uses of magnetic bead purification technology. Comprehensive cDNA libraries (collection of DNA copies of gene-encoding mRNAs that have been cloned into plasmid or virus vectors) could be amplified using biotinylated primers which could then be bound to magnetic beads. These bound cDNAs could then be hybridized to sheared genomic DNA and purified. The genomic DNA could then be released from the bound cDNAs and cloned for conventional sequencing or directly sequenced using 454 technology. A derivation of this technique has utilized microarrays to hybridize and trap sheared genomic DNA that is complimentary to the probes on the array. These trapped sequences can then be stripped off and amplified for 454 or pyrosequencing techniques [11]. 

Sequencing of transcribed regions of the genome is the most common approach for developing genomics data and tools for non-model plants [12]. This is usually accomplished by sequencing cDNAs either by standard Sanger sequencing methods or using newer 454 technologies. Collections of the cDNA sequences are commonly referred to as EST (expressed sequence tags) databases. Because sequences of individual cDNAs can provide an indication of the function of the gene which encoded them, EST databases allow one to identify various genes likely to be involved in specific physiological or developmental processes. This so called “candidate gene approach” serves as a potential staring point to investigate the impact of a given treatment on expression of particular genes of interest [13]. 

Collections of sequenced cDNAs also allow the production of microarrays. Microarrays are glass slides on which thousands of individual cDNAs or fragments of cDNAs (referred to as probes) are spotted in a grid pattern [14]. Fluorescently labeled cDNAs produced from various tissues or following specific treatments can be hybridized to the microarrays. Because the amount of a specific cDNAs that hybridizes to it’s complementary sequences on the array is directly relative to the expression of the gene which encoded them, the brightness of a given spot on the arrays is an indication of the expression level of the gene from which that probe was derived. Since the genes on the array have been characterized, it is possible to draw inferences about the physiological processes that are occurring in a given sample based on the probable function of the genes that are being expressed. However, since sequencing large numbers of ESTs is often not practical in weeds, in some cases, microarrays have been developed from unsequenced cDNAs. In most cases, some or all of the probes from these so called “anonymous arrays” are usually sequenced after they are determined to be of interest by differentially hybridization [15]. Another novel use of microarray technology that has been developed specifically for weedy and non-model systems is the use of commercially available microarrays developed for crop or model plant to follow gene expression in non-model organisms [16-18].

In cases where whole genome sequences are lacking, high density genetic and physical maps compliment and rely on other genomic resources. Simple sequence repeats (SSRs), microsatilites, amplified fragment length polymorphisms (ALFPs), and inter-simple sequence repeats (ISSRs) are polymerase chain reaction (PCR)- based genetic markers that can be developed without prior knowledge of the genomic sequences, and have been a mainstay for researchers working in non-model plants and weeds [19]. The development of high-density genetic maps based on these markers has proven to be of great value for identifying and cloning genes of interest when combined with BAC libraries [20]. Such high density maps can also be used to identify regions of chromosomes that are undergoing evolution in response to altered niches or environments [6]. 

## 2. CURRENT STATE OF GENOMICS RESEARCH IN WEEDS

### 2.1. Comparative Genomics

The use of genomic information for studies in weed science is still in its infancy in most cases. There are currently eight weeds with well developed genomic resources and significant research efforts. Three of these (wild rice, (*Oryza ssp.*), wild sunflowers (*Helianthus ssp.*), and perennial ryegrass (*Lolium perenne*)) have close crop relatives. *Amaranthus* ssp., are related to grain amaranth (*Amaranthus caudatus*) which is a minor crop species in Latin America and was an important crop in the pre-columbian period [21]. The other species leafy spurge (*Euphorbia esua*), dodder (*Cuscuta ssp.*), starthistle (*Centaurea solstitialis*), and spotted knapweed (*Centaurea solstitialis*) do not have close crop relatives. 

### 2.2. Functional Genomics

As yet, the only weedy species for which significant amount of genomic sequence data is available is waterhemp (*Amaranthus tuberculatus*) [22]. 454 technology provided 42 Mbp of sequence data for this weed using only one half of the sequencing capacity of a single experiment. The sequences were aligned into more than 50,000 discrete fragments and included pieces of more than 10,000 different recognizable genes. The number of genes identified in these experiments is surprising since the genome of waterhemp is about 750 Mbps in size. Given the likelihood that most plants have less than 50,000 genes, this single experiment identified sequence fragments for more than a 5^th^ of them. The experiment also generated sequence data for nearly the entire chloroplast genome of this species. Consequently numerous markers, and candidate genes, including genes for herbicide resistance, are now available for waterhemp. The information generated by this pilot study should augment the usefulness of a BAC library resources developed for a related grain amaranth species [23]. 

There are growing numbers of EST databases for numerous weeds including but not limited to leafy spurge, starthistle, and ryegrass, spotted knapweed, and the parasitic weed dodder [24-27]. These databases have provided vast resources for candidate genes used to analyze various physiological processes. For example, Horvath *et al*. 2002 [28], used the leafy spurge EST database to identify molecular markers for cell cycle transitions, and Anderson *et al*. 2005 [29], used the same EST resources to identify several likely sugar metabolism genes to investigate the suspected impact of environmental conditions resulting in dormancy transitions on sugar metabolism in leafy spurge. 

As noted above, EST databases can be used to develop high density microarrays to assess global changes in gene expression (often referred to as transcriptome analysis). However, leafy spurge is the only completely weedy species (i.e. a weed that is not also a crop or ornamental species) for which high density microarrays have been developed [30]. There are high density microarrays available for perennial ryegrass [26], but this species is a common forage crop that can be weedy in some ecosystems. These resources have already been used to provide insight into processes relevant to the biology and invasiveness of these weeds. In ryegrass, transcriptome analysis using microarrays have provided insight into vernalization processes required for perennial growth and disease responses [31, 32]. Both of these traits may influence the invasiveness and fitness of this weed. The leafy spurge microarrays have been used to identify signals and pathways important for dormancy transitions in underground buds, drought responses, and disease resistance [30] [Anderson personal communication] [Santana personal communication]. Anonymous arrays have been used to identify changes in gene expression associated with floral regulation following hybridization between *Senecio* ssps. [33]. 

The earliest reports of using non-weed microarrays to study weed transcriptomes described the use of arabidopsis (*Arabidopsis thaliana*) cDNA arrays and other genomic resources to follow changes in gene expression between shoots and mature leaves of wild oat and leafy spurge [16, 17]. The results from these experiments indicated that hybridization to heterologous arrays could provide useful information on gene expression in a non-model species. More recently, we used cotton microarrays to follow changes in gene expression in velvetleaf (*Abutilon theophrasti*) in response to crop competition [18]. These studies indicated that velvetleaf responded by induction of classic shade avoidance responses. This contrasted to earlier studies using microarray analysis to study how corn responded to velvetleaf [34] which indicated that corn did not exhibit the classic shade avoidance response when competing with velvetleaf. Likewise, Lai *et al*. 2008 [35], used sunflower microarrays to follow differences in gene expression between wild and weedy sunflower species that identified potential changes in gene expression associated with invasiveness. 

There are currently no mutant libraries, such as the T-DNA insertion collection developed for arabidopsis, developed for a weedy species. However, ecotilling techniques have been used in combination with a candidate gene approach to identify mutations in specific genes for a weedy species [36, 37]. In these studies, naturally occurring mutations in the acetolactate synthase gene which is the active site for ALS inhibiting herbicides were identified and catalogued from *Monochoria vaginalis*, a common paddy weed in Japan. This same group used ecotilling techniques to identify naturally occurring mutations in paralogous ALS genes in the same species. These two proof-of-concept papers highlight the potential for utilizing ecotilling to identify mutations in any gene that can be amplified by PCR, even those from polyploid plants. 

The development of mutation libraries through the use of interfering RNAs [38], or insertional-mutagenesis using transposons [39] or agrobacterium derived T-DNA [40] is generally dependent on the ability to transform the target species. Likewise, good transformation systems are needed to confirm hypotheses derived from functional genomics studies. Currently there are only a small number of weeds that have been transformed. These include but are not limited to several weedy *Lolium* ssp. and a related grassy weed, *Agrostis stolonifera* , [41], dodder- *Cuscuta trifolii* [42], horseweed- *Conyza canadensis* [43], *Amaranthus hypochondriacus* [44] and leafy spurge [Chao, personal communications]. 

There are several well developed libraries available for several weedy species. BAC libraries have been produced for grain amaranth [23], and several weedy rice species [45], and are in the process of being developed for leafy spurge. Whole plant normalized libraries used for construction of the leafy spurge EST database, and a similar library developed from Canada thistle (*Cirsium arvense*) have been produced [24] [Anderson, personal communication]. Likewise, we have produced a 2-hybrid capable cDNA library from growing underground buds of leafy spurge, and a lambda zap-based genomic library with average insert size of about 6 kb. The 2-hybird library could be used to identify protein-protein interactions for high throughput proteomics studies. Broz *et al*. 2007 [46], have produced a whole plant normalized cDNA library from spotted knapweed.

### 2.3. Structural Genomics

The use of high density maps and PCR-based markers has been extensively used in wild sunflowers to identify changes associated with interspecies hybridization [47], selection for stress resistance [48], and invasiveness [49]. Because cultivated rice (*Oryza sativa*) has been fully sequenced, and can cross-hybridize to numerous weedy rice species, molecular markers for cultivated rice have been used identify and in some cases clone genes for weedy characteristics such as shattering [50], dormancy [51], and seed color [52]. In addition to these complex studies that have used large numbers of well mapped markers to identify regions of the genome that may be under selection during invasion processes, there are numerous additional examples where a small number of ISSR, AFLP, and microsatellite markers have been used to compare the allelic diversity between invasive and non-invasive weed populations [53-55] among others. 

One of the surprising findings from animal genomics projects is that there appears to be various sizable sections of genomes that have undergone duplication. These duplications have been associated with specific diseases or sub-populations of species, or even in specific tissues within an organism [56]. The discovery and function of the copy number variations (CNV) as they are called is currently a very hot topic in animal genomics. Although CNV has long been associated with transposable elements and complex gene families such as ribosomal RNA genes in plants, there has not been much focus on the type of structural gene amplifications associated with multi-drug resistant cancers, or speciation in mammals. However, recently, there have been as yet unpublished reports of CNV playing a role in herbicide resistance in weeds [57]. Specifically, amplification of the EPSPS gene which is that target site for the herbicide glyphosate appears to be the mechanism responsible for glyphosate resistance of Palmer amaranth (*Amaranthus palmeri*). Given the impact and ubiquity of CNV in animals development, physiology, and evolution, it seems likely that similar processes will be illuminated by genomic studies on these aspects of weeds as well. 

## 3. POTENTIAL FOR GENOMICS RESEARCH IN WEEDS

Although there are limited resources for genomics research in weedy species, there is exceptional potential for utilizing genomic resources developed for crops such as potato (*Solanum tuberosum*), corn (*Zea maize*), and model species such as arabidopsis and poplar (*populus* ssp.). Since many genes are reasonably conserved, it is often possible to use sequence data from model and crop species to amplify and clone candidate genes from non-model and weedy species. Such approaches have been highly successful in identifying herbicide resistance genes from numerous weedy species. For example, the target sites of action for ALS inhibiting herbicides was first cloned from arabidopsis [58]. These sequences were subsequently used to design primers for amplification and cloning homologous genes from many weedy species. In another example, the *SHOOTMERISTEMLESS* gene which is likely involved in production and growth of underground shoot buds on roots of leafy spurge was cloned by identifying conserved sequences from this gene and developing primers that would amplify a portion of the gene which was sued as a probe to isolate the full length gene form cDNA and genomic libraries [59]. Using available sequence databases should readily allow cloning of numerous genes of interest to weed biologists. Many genes involved in altered growth, development, response to stress, etc. have been well characterized in model systems, and are likely to play a role in invasiveness and competitiveness of weeds. 

Fundamental questions of concern to weed scientists such as the evolution and nature of invasiveness and weediness, response to competition and control measures, and origin of weed populations has until recently been unanswerable through conventional studies and techniques. However, the introduction of genomic-based tools and technologies could begin to illuminate the mechanisms and biological processes that make weeds so difficult to control. Plans are underway in several laboratories to use microarray analysis to compare transcriptomes of various invasive weeds in their native and introduced ranges and under common garden conditions to help identify gene expression differences related to invasiveness. Identification of the genes and physiological processes required for weediness and invasiveness should allow the development of methods to help control weeds. Additionally, it is possible that some of the genes that make weeds so competitive may provide targets or information needed to or modify crops and/or management practices to make crops more competitive and able to thrive in less than optimal growing conditions. 
